# Rosiglitazone and Fenofibrate Additive Effects on Lipids

**DOI:** 10.1155/2011/286875

**Published:** 2011-11-24

**Authors:** Ahmad Slim, Laudino Castillo-Rojas, Eddie Hulten, Jennifer N. Slim, Dorette Pearce Moore, Todd C. Villines

**Affiliations:** ^1^Cardiology Service, Brooke Army Medical Center, San Antonio, TX 78234-6200, USA; ^2^Cardiology Service MCHE-MDC, Brooke Army Medical Center, 3851 Roger Brooke Drive, San Antonio, TX 78234-6200, USA; ^3^Cardiology Service, Walter Reed Army Medical Center, Bethesda, MD 20889-5600, USA

## Abstract

*Background*. To evaluate the effect of rosiglitazone, fenofibrate, or their combined use on plasma lipids in normoglycemic healthy adults. *Methods and Results*. Subjects were randomized in a double-blind fashion to rosiglitazone + placebo, fenofibrate + placebo, rosiglitazone + fenofibrate, or matching double placebo. The between-group difference in the change in fasting TG, high-density lipoprotein cholesterol (HDL-C), LDL-C, and plasma apolipoproteins A-I, A-II, and C-III level were compared after 12 weeks of treatment. A total of 548 subjects were screened and 41 met the inclusion criteria. After 12 weeks of therapy, the median change in the triglyceride levels showed a significant reduction ranging from 47 to 55 mg per deciliter in the fenofibrate only and rosiglitazone/fenofibrate groups compared with placebo (*P* = 0.0496). However, the rosiglitazone only group did not show significant change in triglyceride level. The change in the Apo AII showed increase in all the treatment groups compared with placebo (*P* = 0.009). There was also significant change in the Apo CIII that showed reduction of its level in the fenofibrate only and rosiglitazone/fenofibrate groups (*P* = 0.0003). *Conclusion*. Rosiglitazone does not appear to modulate hypertriglyceridemia in patients with elevated triglycerides independent of glucose metabolism.

## 1. Background

The thiazolidinediones (TZDs), which include troglitazone (withdrawn by the FDA), rosiglitazone, and pioglitazone, correct hyperglycemia in diabetic patients by increasing insulin sensitivity in both the liver [1, 2] and skeletal muscles [3, 4]. The mechanism involved in the plasma lipid and lipoprotein changes induced by thiazolidinediones (TZDs) remains unclear. It is possible that these agents indirectly alter plasma lipid and lipoprotein levels indirectly by improving insulin sensitivity and glycemic control or directly by influencing lipoprotein synthesis and/or catabolism. The intent of this study is to assess whether rosiglitazone affects lipids independent of glycemic control by testing the hypothesis in normoglycemic patients with elevated TG. The presence of synergistic effect when combined with fenofibrate will be evaluated as well.

Clinical trials using TZDs in type 2 diabetic subjects have observed that these agents also favorably impact plasma lipid and apolipoprotein concentrations. Following eight weeks of treatment with rosiglitazone (4 mg, twice daily) in 243 type 2 diabetic patients, the mean HDL-C increased by 6% and TG by 2%. The increase in the LDL-C concentration (9%) was accompanied by a shift in small, dense LDL to large, buoyant LDL in 52% of the treated subjects. The shift in LDL size occurred independent of a significant triglyceride reduction, which is in contrast to several studies reporting that increases in LDL size are significantly correlated with a decrease in the plasma concentrations of total and very-low-density lipoproteins (VLDL) and triglycerides [5, 6]. The mechanism involved in the plasma lipid and lipoprotein changes induced by TZDs remains unclear. It is possible that these agents indirectly alter plasma lipid and lipoprotein levels indirectly by improving insulin sensitivity and glycemic control or directly by influencing lipoprotein synthesis and/or catabolism.

Three distinct peroxisome proliferator-activated receptors (PPARs), termed alpha, beta, and gamma, modulate intracellular lipid and glucose metabolism through controlling gene expression when activated [7]. Activation of PPAR-alpha leads to decrease production of ApoC-III, which in turn increases the synthesis of lipoprotein lipase, and triglyceride catabolism. Gene expression for the synthesis of ApoA-I and ApoA-II is also enhanced by activation of PPAR-alpha, resulting in increase in HDL concentration. Fibric acid derivatives (gemfibrozil and fenofibrate) reduce triglycerides and increase HDL-C by binding to the PPAR-alpha nuclear receptor. Theoretically, TZDs given to nondiabetic individuals should not modulate lipid through its effect on PPAR-gamma, and any change would be anticipated to be due to binding to PPAR-alpha. Thus, it would result in decrease in the plasma concentration of ApoC-III and an increase in ApoA-I and ApoA-II, with a subsequent rise in HDL-C and reduction in triglyceride concentration.

The Rosiglitazone and Fenofibrate Additive Effects on Lipids (RAFAEL) trial was designed to evaluate the effect of rosiglitazone when combined with fenofibrate on the plasma lipid and lipoprotein concentrations assuming direct influence on the synthesis of the apolipoproteins that are responsible for VLDL and HDL metabolism in normo-glycemic individuals with elevated TG as well as the mechanism of action of rosiglitazone.

## 2. Methods

### 2.1. Study Protocol and Oversight

The Rosiglitazone and Fenofibrate Additive Effects on Lipids (RAFAEL) protocol was reviewed and approved by the institutional review board at Brooke Army Medical Center where the trial was conducted. The study was sponsored by GlaxoSmithKline (London, UK), ClinicalTrials.gov ID: NCT00819910.

### 2.2. Study Population

All patients provided written informed consent to participate in the study. Patients were eligible if they were 18 years of age or older, had a fasting glucose <100 mg/dL, fasting LDL-C <160 mg/dL, and a triglyceride <400 mg/dL. Patients taking any cholesterol lowering medication prior to entering the study underwent a “wash-out” period of two weeks. Patients were excluded if they had a history of congestive heart failure, evidence of renal impairment (Cr > 1.4 mg/dL), history of liver disease (ALT and/or AST above the upper level of normal), known diabetes mellitus or impaired fasting glucose (fasting glucose > 100 mg/dL), pregnant or breast feeding, prior history of an acute coronary syndrome, myocardial infarction, or coronary revascularization, life-threatening disease with an estimated survival of less than 3 years, or inability to take rosiglitazone and/or fenofibrate.

### 2.3. Study Design

At the beginning of the trial, candidates were instructed to fast for 12 to 15 hours for the initial visit. Baseline fasting lipid profile with direct LDL-C measurement, fasting glucose, hepatic function and plasma concentrations of apolipoproteins A-I, A-II, and C-III were drawn at the initial visit. Based on the concentration of total cholesterol, triglyceride, and glucose, eligible subjects were randomized within 7 days of providing the initial blood sample. Subjects, who met the inclusion criteria, were then randomized to one of 4 groups: rosiglitazone (dose 8 mg daily) plus placebo; fenofibrate (145 mg daily) plus placebo; fenofibrate (145 mg daily) plus rosiglitazone (dose 8 mg daily); or double placebo for a total of 12 weeks. At the mark of 12 weeks (final visit), the initial laboratory collections were repeated to assess the difference in concentration of fasting glucose, insulin, hepatic transaminases, fasting lipid profile, apolipoproteins, HDL size, LDL size, and statistical difference from baseline. At this visit, subjects were asked to return their bottles for a pill count to assess compliance and assessment of adverse events was done at this time. All blood samples were shipped to Oklahoma Research Foundation for apolipoproteins A-I, A-II, and C-III assessments and the remainder of the labs were shipped to Quest Diagnostic Labs.

### 2.4. End Points

The primary end-point with respect to efficacy was the between-group difference in the change in TG levels after 12 weeks of treatment. Secondary end-points included the between-group difference in the change in HDL-C, LDL-C, Apo AI, Apo AII, and Apo CIII levels. The primary safety end-point was the incidence of elevations in AST and ALT, defined as more than three times the upper limit of normal.

### 2.5. Statistical Analysis

The null hypothesis was that there will be no difference in the serum triglyceride level between treatments. The alternative hypothesis is that there will be at least a 20% decrease in the serum triglyceride level in the fenofibrate, rosiglitazone, fenofibrate, and rosiglitazone treatment groups relative to the placebo group.

The sample size was determined to be 16 subjects in each arm (64 subjects total) based on eight comparisons between groups before and after treatment with 80% power and a 95% level of confidence. Anticipating a 25% drop out rate, aimed for 20 subjects per group for a total of 80 subjects, but we were only able to recruit 41 subjects due restrictions to the prescriptive pattern with thiazolidinediones per the Food and Drug Administration that occurred after the start of the trial. Statistical differences among the four treatment groups were assessed by comparing the median changes among groups using Kruskal-Wallis one-way analysis of variance on ranks. *P* values < 0.05 were considered statistically significant.

## 3. Results

Between October 2008 and August 2010, a total of 548 patients were screened at one center. Of the 548 patients who were screened, a total of 41 patients met the inclusion criteria and were randomly assigned one of the prespecified study groups. As of October 2010, follow-up assessment, with ascertainment of end points, was completed in 73% of the patients in the study. There were seven (17%) patients that did not complete the study due to either lost of followup or adverse events that make them to withdraw from the study. The baseline characteristics of the patients who were enrolled in the study are shown in Table 1. The mean age was 56 years; 48% of the patients were male, and 39% of the patients were treated for hypertension. At baseline, the mean total cholesterol was 214 mg per deciliter, mean HDL cholesterol was 48 mg per deciliter, mean LDL cholesterol was 121 mg per deciliter, and mean triglyceride was 240 mg per deciliter.

**Figure 1:**
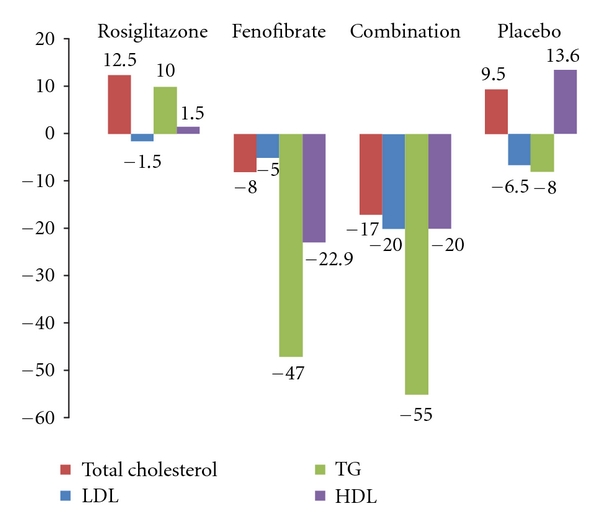
Median change in lipid profile after treatment.

**Figure 2:**
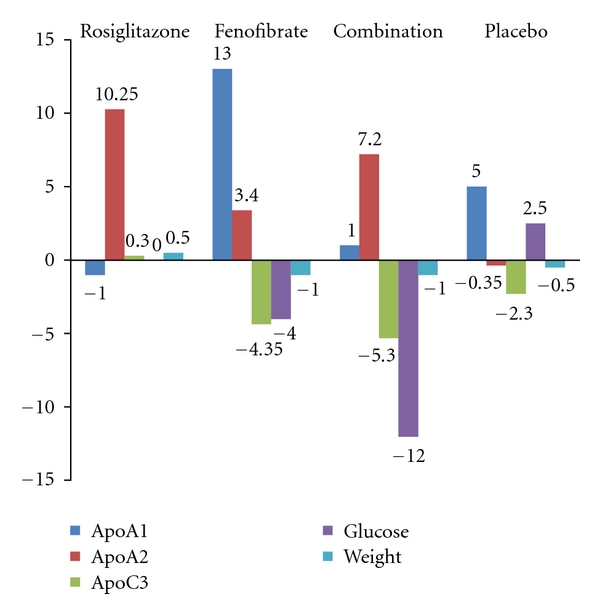
Median change before and after treatment in Apo subparticle, blood sugar, and weight.

After 12 weeks of therapy, the change in the triglyceride levels showed a significant reduction ranging from 47 to 55 mg per deciliter in the fenofibrate only and rosiglitazone/fenofibrate groups compared with placebo (*P* = 0.0496) (Figure 1). However, the rosiglitazone only group did not show significant change in triglyceride level. There was also a significant reduction in the HDL levels showing a significant reduction ranging from 20 to 22 mg per deciliter compared with placebo (*P* = 0.0152) (Figure 1). There was no significant difference in the median change in the total cholesterol or LDL cholesterol between the groups (Figure 1). In regards to the apolipoproteins, the median change in the Apo AII showed a significant increase in all the treatment groups compared with placebo (*P* = 0.009) (Figure 2). There was also significant median change in the Apo CIII that showed reduction of its level in the fenofibrate only and rosiglitazone/fenofibrate groups (*P* = 0.0003) (Figure 2). There was no difference in median change in Apo AI (Figure 2).

### 3.1. Safety End Points

There were adverse events reported in seventeen patients in the study. Four adverse events led to discontinuation of therapy in most cases during the study (three ADE's in the combined arm and one in the double placebo arm). There was one patient in the combined (rosiglitazone and fenofibrate) group who was hospitalized during the study due to acute renal failure (SAE's). The most common adverse effects were headache and gastrointestinal complains such as abdominal discomfort, diarrhea, nausea, and vomiting. The level of transaminases did not increase significantly in any of the patients in each treatment group. 

## 4. Discussion

Clinical trials have shown that the use of thiazolidinedione in type 2 diabetic patients has shown favorable impact in plasma lipid and lipoprotein concentrations in addition to glycemic control. The mechanism involved in the plasma lipid and lipoprotein concentration changes in type 2 diabetics by thiazolidinedione remains unclear. The RAFAEL trial is the first study performed in the literature to evaluate the effect of thiazolidinedione to plasma lipid and apolipoprotein concentration in normoglycemic population to assess mechanism of action. After 12 weeks of therapy, the median change in the triglyceride level was only significant in the group that received fenofibrate as expected due to its known mechanism of action. We also saw significant changes in the fenofibrate/rosiglitazone group which were most likely due to the fenofibrate alone since it was very similar change compared to the fenofibrate alone. In addition, the rosiglitazone group did not show a significant change in the triglyceride level. Therefore, combination therapy of rosiglitazone and fenofibrate did not show trend towards additive reduction in triglyceride levels.

In regards to the apolipoproteins, all treatment groups showed significant increase in the median change of Apo AII compared to placebo. Also, median change of Apo CIII showed significant reduction of its level in the fenofibrate only and fenofibrate/rosiglitazone groups. However, this change was not seen in the rosiglitazone group only. As a result, rosiglitazone does not affect Apo CIII levels and subsequently lipoprotein lipase activity as anticipated. However, rosiglitazone increases Apo AII levels without observed change in HDL-C in patients with type 4 hyperlipidemia with normoglycemia. Fibrates impact on TG reduction is known to correlate with its reduction of Apo CIII in animal models [8]. The absence of impact on ApoC III and TG simultaneously by Rosi does not negate the fact that it might bind to PPAR alpha and therefore affecting ApoAII, but it negates its independent impact on lipid metabolism as a PPAR-alpha strong agonist independent of glucose metabolism.

Several limitations of the current study should be considered. First, the study was terminated early per FDA recommendations restricting rosiglitazone use. Second, recruitment of patients was slow due to deployment restraints and the publication of various studies evaluating the cardiovascular complications of rosiglitazone. Therefore, the sample size was insufficient for power analysis, and strength of statistical significance was adversely affected in all end points.

## 5. Conclusion

In conclusion, the results from our study suggest the favorable effects of rosiglitazone on plasma lipid and lipoprotein concentrations in diabetic patients noted in prior studies are not independent of glucose control as noted in our normoglycemic cohort of subjects.

## Figures and Tables

**Table 1:** Baseline characteristics and laboratory findings with posttreatment changes.

Group	Placebo	Fenofibrate	Rosiglitazone	Combined	ANOVA
	*N* = 10	*N* = 9	*N* = 8	*N* = 7	Rank
	Mean ± SD	Mean ± SD	Mean ± SD	Mean ± SD	*P*-value
Age	57.4 ± 11.1	61.2 ± 11.6	57.3 ± 8.4	54.7 ± 9.5	
M : F	6 : 4	3 : 6	5 : 3	3 : 4	

Pre-TG	206 ± 65	278 ± 126	234 ± 58	239 ± 73	*P* = 0.212
Post-TG	202 ± 53	192 ± 64	240 ± 115	172 ± 27	
(%Δ)	(7.6% ± 51.0%)	(−2.2% ± 26.0%)	(7.4% ± 48.9%)	(20.0% ± 36.5%)	

Pre-HDL-C	48 ± 9	41 ± 8	52 ± 19	52 ± 14	*P* = 0.342
Post-HDL-C	48 ± 9	47 ± 7	50 ± 17	53 ± 9	
(%Δ)	(1.7% ± 10.5%)	(14.5% ± 21.6%)	(1.9% ± 24.6%)	(5.8% ± 16.4%)	

Pre-LDL-C	124 ± 46	111 ± 40	145 ± 24.6%	106 ± 41	*P* = 0.692
Post-LDL-C	128 ± 39	118 ± 32	140 ± 41	102 ± 31	
(%Δ)	(13.7% ± 47.8%)	(2.6% ± 29.3%)	(−.5% ± 27.4%)	(37.3% ± 141.6%)	

± pre-Tot C	204.4 ± 57.9	213.6 ± 39.0	238.4 ± 39.5	215.7 ± 36.6	*P* = 0.372
Post-Tot C	227.4 ± 37.2	199.0 ± 51.2	230.9 ± 52.2	201.1 ± 36.5	
(%Δ)	(25.8% ± 70.2%)	(−7.7% ± 26.1%)	(−2.3% ± 21.3%)	(−5.0% ± 21.6%)	
